# MAPK Pathway Activation Patterns in the Synovium Reveal ERK1/2 and EGFR as Key Players in Osteoarthritis

**DOI:** 10.3390/biomedicines13122992

**Published:** 2025-12-05

**Authors:** Ivana Jurić, Petar Todorović, Nela Kelam, Danica Boban, Patricija Bajt, Anita Racetin, Matko Rošin, Davor Čarić, Katarina Vukojević

**Affiliations:** 1 Department of Emergency Medicine, University Hospital of Split, Spinciceva 1, 21000 Split, Croatia; ivana.juric@zhmsdz.hr; 2 Department of Anatomy, Histology and Embryology, University of Split School of Medicine, Šoltanska 2A, 21000 Split, Croatia; petar.todorovic@mefst.hr (P.T.); nela.kelam@mefst.hr (N.K.); danica.boban@mefst.hr (D.B.); patricija.bajt@mefst.hr (P.B.); anita.racetin@mefst.hr (A.R.); 3 Department of Surgery, Orthopaedics and Traumatology Division, University Hospital of Split, Spinčićeva 1, 21000 Split, Croatia; mrosin@kbsplit.hr (M.R.); dcaric@kbsplit.hr (D.Č.); 4 Center for Translational Research in Biomedicine, University of Split School of Medicine, Šoltanska 2A, 21000 Split, Croatia; 5 Mediterranean Institute for Life Sciences, University of Split, Meštrovićevo Šetalište 45, 21000 Split, Croatia

**Keywords:** osteoarthritis, synovial membrane, ERK 1/2, p38, JNK, EGFR, MAPK

## Abstract

**Background/Objectives**: Chronic synovitis is a hallmark of osteoarthritis (OA) progression, driving cartilage degradation via inflammatory mediators. While the MAPK signaling pathway is implicated in OA pathogenesis its activation patterns in hip synovium remain poorly characterized, and regional differences within the synovial membrane have not been systematically examined. This research aims to determine the expression of extracellular signal-regulated kinase 1/2 (ERK1/2), p38 mitogen-activated protein kinase (p38 MAPK), c-Jun N-terminal kinase (JNK), and the Epidermal Growth Factor Receptor (EGFR) in the MAPK signaling pathway in the synovial membrane of osteoarthritic hips. **Methods**: We compared synovial immunofluorescence expression of the aforementioned proteins in a control (CTRL) group of subjects with femoral neck fractures and a group with hip OA. **Results**: Higher ERK1/2 immunoexpression was detected in the intima compared with the subintima in the CTRL group (*p* < 0.05), and a similar distribution was observed in the OA group (*p* < 0.0001). The intima of the OA group exhibited a considerably greater area percentage of positive signal than the intima of the CTRL group (*p* < 0.01). In all groups examined, we observed that p38 MAPK expression was markedly more positive in the intima than in the subintima (*p* < 0.0001), but without statistically significant differences between groups. JNK and EGFR immunoexpression were higher in the intima than in the subintima across all analyzed groups, but the difference did not reach statistical significance (*p* > 0.05). No differences in the expression of these two markers were detected between the CTRL and OA groups (*p* > 0.05). Differential analysis of the GEO dataset revealed no significant differences in expression between the OA and CTRL groups in the expression of *MAPK1*, *MAPK3*, *MAPK8*, *MAPK9*, *MAPK10*, and *MAPK11. EGFR* was significantly elevated in OA compared to CTRLs in the differential analysis of the GEO dataset. **Conclusions**: This study provides the first comprehensive analysis of MAPK pathway activation in hip OA synovium, revealing ERK1/2 as a key player with region-specific upregulation in the synovial intima. Combined with elevated EGFR expression, these findings suggest potential therapeutic targets for hip OA synovitis. The discordance between protein and mRNA levels for ERK1/2 indicates post-transcriptional regulation, warranting further investigation into phosphorylation status and functional activation. Our results support the development of targeted interventions for hip OA, a condition with limited treatment options beyond joint replacement.

## 1. Introduction

Osteoarthritis (OA) is a chronic degenerative disease characterized by joint pain and functional disability that can involve every joint in the body. OA can be divided into primary, with an unknown underlying cause, and secondary, caused by another condition [1,2]. The most significant hallmark of OA is chronic synovitis, which leads to the progressive degradation and loss of joint cartilage, resulting in abnormal bone remodeling [3]. The knee and hip joints are mainly affected by this most common form of arthritis, which reduces joint function and thus impairs quality of life [4,5,6]. Being the most prevalent form of arthritis, OA affects approximately 300 million individuals worldwide [7], and risk factors involve age, adiposity, genetics, female gender, obesity, articular injury, and excessive loading of the joints [8].

The mitogen-activated protein kinase (MAPK) cascade signaling pathway is believed to contribute to the development and progression of hip OA [9]. The MAPK cascade is a signaling pathway consisting of a three-tiered system of protein kinases essential for cellular homeostasis [10,11]. It is particularly involved in gene expression, control of cellular behavior, cell proliferation, inflammatory cell response, and cell death. Abnormal activation of the MAPK signaling pathway leads to chondrocyte dysfunction, enhanced inflammatory response in cartilage tissue, and promotes tissue degradation [12,13,14,15]. In OA, increased MAPK pathway activity in synovial tissue amplifies inflammation and cartilage destruction through mediators such as IL-1, TNF-α, growth factors, and matrix metalloproteinases (MMP-1, MMP-3, MMP-13), ultimately leading to chondrocyte apoptosis and progressive joint damage [16,17,18,19,20,21].

Given the crucial role of MAPK signaling in inflammation and cartilage degradation, particular attention has been directed to its key components—extracellular signal-regulated kinase 1/2 (ERK1/2), p38 mitogen-activated protein kinase (p38 MAPK), c-Jun N-terminal kinase (JNK), and Epidermal Growth Factor Receptor (EGFR) —which have been implicated in OA progression. By comparing their expression levels in synovial tissue from patients with OA and control subjects, this study aims to further elucidate their contribution to OA pathogenesis.

ERK1/2 is a member of the MAPK family that regulates chondrocyte proliferation, hypertrophic differentiation, cartilage calcification, and osteophyte formation [12,22,23]. In osteoarthritic conditions, phosphorylation of ERK1/2 together with reduced p38 activity promotes hypertrophic differentiation of articular chondrocytes through interactions with subchondral bone osteoblasts [24]. Activation of the ERK1/2 signaling pathway by pro-inflammatory cytokines enhances MMP expression and activity in articular chondrocytes, contributing to cartilage degradation [25]. Moreover, IL-1β-induced phosphorylation of ERK1/2 activates Dynamin-related protein 1 (DRP1), a key mediator of mitochondrial network fragmentation and chondrocyte apoptosis, which has been proposed as a potential therapeutic target in OA [26].

The p38 MAPK contributes to cartilage damage in OA by promoting inflammatory responses and inducing apoptosis of articular chondrocytes [27,28,29,30]. Both p38 MAPK and ERK1/2 mediate IL-1-induced downregulation of aggrecan, a key component of the extracellular matrix that, together with type II collagen, ensures tissue structural stability and supports chondrocyte differentiation [27,31]. Inhibition of the p38 MAPK pathway has been shown to reduce OA progression by decreasing the expression of pro-inflammatory cytokines such as IL-1β and TNF-α [32,33,34]. Additionally, microRNAs, including miR-124 and miR-296-5p, modulate OA pathogenesis by targeting genes regulated by p38 MAPK [34,35].

JNK, a serine/threonine MAPK, regulates gene expression related to inflammation, apoptosis, and cell proliferation through phosphorylation of target proteins [36,37]. Although its role in chondrogenesis is less prominent than that of ERK or p38/SAPK pathways, JNK is activated by cellular stress and pro-inflammatory cytokines, such as IL-1 and TNF, contributing to MMP expression, extracellular matrix degradation, and cartilage destruction [38,39,40,41]. In mouse models, JNK2 mediates IL-1-dependent aggrecan degradation and promotes OA progression by increasing MMP-13 production while reducing proteoglycan synthesis. Furthermore, JNK signaling is active in OA synovial membranes and mediates adiponectin-induced ICAM-1 expression, facilitating monocyte adhesion and inflammation [42,43].

EGFR is a cell surface tyrosine kinase receptor expressed in both healthy and OA cartilage [44,45,46]. In healthy cartilage, it promotes chondrocyte proliferation, survival, and matrix production while inhibiting apoptosis [44,47]. In OA, EGFR overexpression increases MMP production and elevates inflammatory mediators in the synovial membrane [47,48]. Studies in mouse models indicate that EGFR is essential for cartilage homeostasis: partial inhibition slows cartilage degradation, whereas complete loss exacerbates OA progression [47].

Despite extensive research on MAPK signaling in osteoarthritis, several critical gaps remain in our understanding of its role in synovial inflammation, particularly in hip OA. First, the vast majority of studies have focused on knee OA synovium, with limited investigation of MAPK pathway activation in hip synovial tissue, which exhibits distinct biomechanical loading patterns and inflammatory characteristics compared to the knee joint. Second, while previous studies have characterized MAPK expression in bulk cartilage or whole synovial tissue, regional differences between the synovial intimal and subintimal layers have not been systematically examined, despite their functional specialization in inflammation and tissue remodeling. Third, most prior work has relied on either protein-level or gene expression analyses in isolation, without integrating both approaches to validate findings across multiple analytical platforms.

The present study addresses these gaps by characterizing the expression patterns of ERK1/2, p38 MAPK, JNK, and EGFR specifically in hip OA synovium, with detailed analysis of their regional distribution between the intima and subintima. We combine quantitative immunofluorescence analysis of protein expression with differential gene expression analysis from publicly available transcriptomic datasets (GEO), enabling cross-validation of findings and distinguishing between transcriptional and post-transcriptional regulation. Furthermore, we correlate MAPK pathway activation with histopathological severity, as assessed by the Krenn synovitis score, providing a clinically relevant context for the molecular findings.

Our results identify ERK1/2 and EGFR as differentially expressed MAPK pathway components in hip OA synovium, with distinct intimal predominance, suggesting their potential as therapeutic targets or biomarkers for synovial inflammation. These findings expand our understanding of joint-specific mechanisms in OA pathogenesis and may inform the development of targeted interventions for hip OA, a condition with limited non-surgical treatment options.

## 2. Materials and Methods

### 2.1. Study Population

The study was conducted in accordance with the Declaration of Helsinki and approved by the Ethics Committee of the University Hospital Split, Croatia, on 27 November 2023 (protocol 500-03/23-01/230). It consisted of two parts: the clinical phase, executed at the Division of Orthopedics and Traumatology, which involved patient selection, surgical collection of hip tissue samples, and tissue processing, and the experimental phase, conducted at the Department of Anatomy, Histology, and Embryology, which involved histological and immunofluorescence staining, as well as data collection and image analysis. All participants provided written informed consent to participate in the study.

A total of 34 subjects were included in this study, with 24 in the OA group and 10 in the control (CTRL) group. Subjects in the OA group were diagnosed with hip OA based on clinical and radiological criteria and had undergone long-term conservative treatment without improvement in joint function or pain. OA diagnosis and inclusion were further confirmed using the Harris Hip Score (HHS), Western Ontario and McMaster Universities Arthritis Index (WOMAC), visual analog scale (VAS), and the Kellgren–Lawrence (K-L) radiological grading scale. Exclusion criteria for the OA group included hip dysplasia, rheumatic diseases, previous hip fractures, and prior hip infections.

The CTRL group consisted of patients undergoing hip arthroplasty for femoral neck fractures, with absent radiographic evidence of OA (Kellgren–Lawrence grade 0–1), no documented history of rheumatic or infectious hip disease, and negative serology for anti-cyclic citrullinated peptide (anti-CCP) and rheumatoid factor (RF). Clinical, radiological, and histopathological characteristics of both groups are presented in Table 1.

### 2.2. Tissue Collection and Basic Staining Procedures

The patients were scheduled for total hip arthroplasty, performed under spinal anesthesia, during which a posterolateral surgical approach was used to incise the small rotators and posterior capsule, followed by joint luxation. Using a triangular oscillating saw (Trauma Recon System, Synthes, Oberdorf, Switzerland), the Synovial tissue showing the highest degree of damage in the femoral load-bearing (the lower part of the femoral neck near the femoral head) area was collected. Synovial tissue samples from both groups were evaluated according to Krenn’s synovitis score [49] and are presented in Table 1.

The tissue was subsequently processed and embedded in paraffin. At the Department of Anatomy, Histology, and Embryology, it was sectioned into 5 µm slices using a rotary microtome (RM2125 RTS, Leica, Buffalo Grove, IL, USA) for further staining and analysis, as previously described [49,50,51]. Appropriate tissue preservation was confirmed through hematoxylin–eosin (H&E) staining of every tenth slide.

### 2.3. Immunofluorescence Staining

The immunofluorescence procedure followed the methodology described in earlier reports [52,53,54]. Histological sections were first deparaffinized in xylene, rehydrated through graded ethanol solutions (100%, 96%, 70%), and rinsed in distilled water. Antigen retrieval was performed by heating the slides for 30 min at 95 °C in 0.01 M citrate buffer (pH 6.0), followed by cooling to room temperature and washing with 0.1 M phosphate-buffered saline (PBS). Sections were then incubated in protein blocking solution (ab64226, Abcam, Cambridge, UK) for 20 min to prevent nonspecific staining. Primary antibodies (Table 2) were applied and incubated overnight in a humid chamber (StainTray slide staining system; Sigma-Aldrich, St. Louis, MO, USA) at room temperature. After PBS washes, sections were incubated for 1 h with secondary antibodies (Table 2), followed by a final PBS wash and nuclear staining with DAPI (4′,6-diamidino-2-phenylindole). Slides were mounted with Immu-Mount (Thermo Shandon, Pittsburgh, PA, USA) and covered with a glass coverslip [49,50,51].

To ensure specificity and minimize nonspecific background, isotype-matched, secondary-only, and positive controls were included. In isotype-matched controls, the primary antibody was replaced with a non-target antibody of the same isotype to assess nonspecific binding. Secondary-only controls omitted the primary antibody to identify any nonspecific interactions of the secondary antibody. Positive controls were used to confirm the validity and reliability of the staining procedure.

### 2.4. Data Acquisition and Quantitative Analysis

H&E slides were examined using a bright-field light microscope (CX43, Olympus, Tokyo, Japan). Immunofluorescent-stained slides were reviewed and microphotographs captured using a fluorescent microscope (Olympus BX51, Tokyo, Japan) equipped with a Nikon DS-Ri2 camera (Nikon Corporation, Tokyo, Japan) with NIS-Elements F software (version 5.22.00). Ten non-overlapping fields per sample were photographed at 40× objective magnification, and the images were analyzed for positively stained proteins (ERK1/2, p38 MAPK, JNK, and EGFR), with positive signals shown in green. For each captured microphotograph, we calculated the percentage (%) of the area covered by positive signal for quantitative analysis of each protein. Separation of the synovial intima from the subintima was performed in Adobe Photoshop (Adobe, San Jose, CA, USA) using the Lasso tool. Positive signal isolation and analysis were conducted in ImageJ (version 1.54, NIH, Bethesda, MD, USA) as previously described [51,52,55,56]. Image processing was performed using the triangle method, and the percentage of stained area was quantified with the “Analyze Particles” function. To account for tissue deficits in some images, the percentage area was corrected by dividing the uncorrected percentage multiplied by the total number of pixels minus the number of empty space pixels.

#### 2.4.1. Synovial Lining Thickness Measurement

Synovial lining thickness was measured using ImageJ software. For each tissue section, ten perpendicular measurements from the synovial surface to the sublining layer were obtained at evenly spaced intervals across the tissue. Scale calibration was performed using the embedded scale bar from microscope images (1 pixel = 0.454 µm at 200× magnification). Measurements were performed by two independent observers blinded to patient group assignment. Inter-observer reliability was assessed using the intraclass correlation coefficient (ICC = 0.91, 95% CI: 0.86–0.95).

#### 2.4.2. Cellularity Quantification

Sublining cellularity was quantified by manual counting of hematoxylin-positive nuclei within standardized regions of interest (ROI, 0.1 mm^2^ = 220 µm × 450 µm) positioned in the sublining layer immediately beneath the synovial lining. Five non-overlapping ROIs were analyzed per tissue section, avoiding areas with blood vessels or adipose tissue. Cell density was expressed as cells per mm^2^. Nuclear profiles with a diameter <3 µm were excluded to prevent counting debris or apoptotic bodies.

### 2.5. Statistical Analysis

Statistical analyses were performed using GraphPad Prism version 9.0.0 (GraphPad Software, San Diego, CA, USA). Data are presented as mean percentages ± standard deviations (SD) for continuous variables and median with interquartile range for ordinal scores. Data normality was assessed using the Shapiro–Wilk test. Because the data were not normally distributed, the Kruskal–Wallis test was used to compare clinical, radiological, and histopathological characteristics between groups.

Quantitative histological parameters, including synovial lining thickness (µm) and sublining cellularity (cells/mm^2^), were compared between the control and OA groups using an unpaired *t*-test with Welch’s correction for unequal sample sizes and variances.

Differences in protein expression between sample groups were analyzed using two-way analysis of variance (ANOVA) followed by Tukey’s post hoc test. Statistical significance was defined as *p* < 0.05.

### 2.6. Differential Gene Expression Analysis

The Gene Expression Omnibus (GEO) database, maintained by the National Center for Biotechnology Information (NCBI), provides access to a wide range of gene expression datasets [57]. To identify datasets relevant to our study, we searched GEO using the keywords “osteoarthritis”, “Homo sapiens”, and “Expression profiling by array”, which returned 114 studies. From these, we selected the GSE55235 series (Rheumatoid arthritis and osteoarthritis: synovial tissues—Berlin dataset [58]), which includes gene expression data from 30 synovial tissue samples: 10 from osteoarthritic joints, 10 from rheumatoid arthritis (RA) joints, and 10 control samples from healthy joints matched for age and gender [58]. For our analysis, we focused on the OA and healthy CTRL groups, excluding RA samples, as they were outside the scope of this study. Total RNA (3–5 µg) was used for amplification and labeling with GeneChip^®^ one-cycle target labeling and control reagents (Affymetrix). Hybridization was performed on GeneChips and scanned using the Affymetrix GeneChip Scanner 3000 (Affymetrix^®^ Inc., Santa Clara, CA, USA). Raw gene expression data were analyzed using GEO2R, an online statistical analysis tool provided by NCBI [57]. Adjusted *p*-values were calculated using the Benjamini–Hochberg method (false discovery rate). The data were processed using the limma package (version 3.28.14), employing vooma (limma precision weights) and quantile normalization. Differentially expressed genes (DEGs) were identified based on the following criteria:|log_2_ (fold change)| > 1 and *p* < 0.05. Genes with log_2_FC ≥ 1 were considered upregulated, while those with log_2_FC ≤ −1 were considered downregulated. The volcano plot was obtained from the same dataset and subsequently edited using Adobe Photoshop (version 21.0.2).

We also evaluated GSE82107 (*n* = 10 OA, *n* = 7 controls); however, this dataset yielded inconsistent results across studies, with reported DEGs ranging from 8 to over 3000 depending on analytical parameters [59,60]. Therefore, GSE55235 was selected as the primary dataset for transcriptomic validation.

### 2.7. KEGG Pathway Enrichment Analysis

To identify biological pathways significantly enriched among differentially expressed genes in OA synovial tissue, KEGG (Kyoto Encyclopedia of Genes and Genomes) pathway enrichment analysis was performed using ShinyGO v0.77 (http://bioinformatics.sdstate.edu/go/, accessed on 21 July 2025) [61]. The complete list of 1356 differentially expressed genes (DEGs) identified from the GSE55235 dataset (|log_2_(fold change)| > 1 and *p* < 0.05) was submitted to the ShinyGO web interface with Homo sapiens selected as the reference organism.

Pathway enrichment analysis was conducted using the hypergeometric distribution test to assess the statistical overrepresentation of DEGs in predefined KEGG pathways relative to the background gene set. *p*-values were adjusted for multiple testing using the Benjamini–Hochberg false discovery rate (FDR) correction method. KEGG pathways with FDR < 0.05 were considered significantly enriched. For each enriched pathway, the following parameters were recorded: (1) number of DEGs mapped to the pathway (nGenes); (2) total number of genes annotated to the pathway in the KEGG database (Pathway Genes); (3) fold enrichment calculated as (nGenes/total DEGs)/(Pathway Genes/total genome genes); (4) adjusted *p*-value (FDR).

Results were visualized as a horizontal bar plot displaying pathway names ranked by fold enrichment, with bar length representing fold enrichment values and color gradient indicating the magnitude of enrichment (red for highest enrichment, blue for lowest enrichment among significant pathways). The MAPK signaling pathway was explicitly highlighted for its relevance to the study objectives.

## 3. Results

### 3.1. Histopathological Features of Synovial Tissue in Osteoarthritis

To establish the histological context for subsequent molecular analyses, we first characterized the architectural features of synovial tissue obtained from control (*n* = 10) and OA (*n* = 24) patients undergoing total hip arthroplasty.

#### 3.1.1. Normal Synovial Architecture in Control Tissue

**Figure 1:**
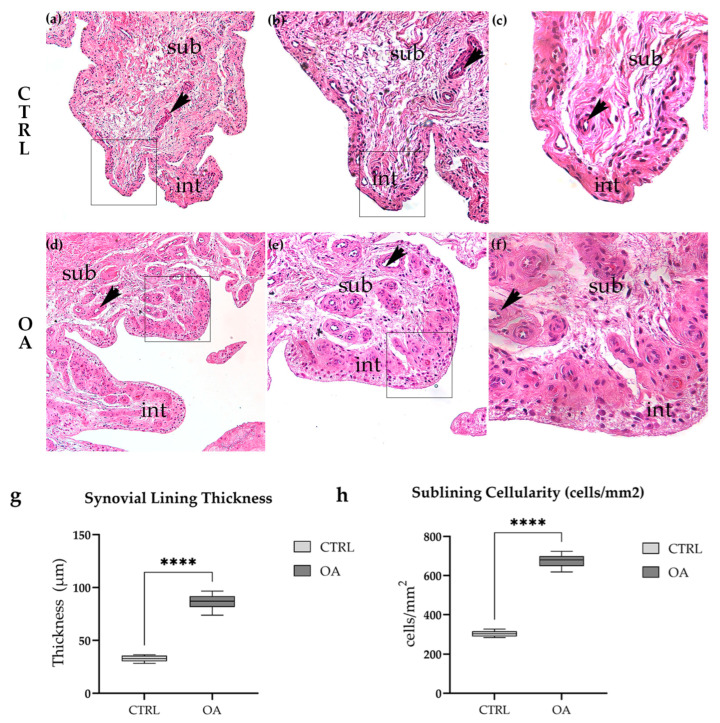
Histological examination of H&E-stained synovial tissue revealed marked architectural differences between control and OA samples. Control synovium (**a**–**c**) exhibited normal architecture with a thin intimal lining (1–2 cell layers) and loose sublining containing scattered fibroblasts and adipocytes. In contrast, OA synovium (**d**–**f**) demonstrated characteristic features of chronic synovitis, including synovial lining hyperplasia, increased cellularity, inflammatory cell infiltration, and evidence of neovascularization. Patient demographics for representative images: CTRL (**a**–**c**): 64F, BMI 24.2, K-L 0; OA (**d**–**f**): 69F, BMI 28.1, K-L III. (**a**–**f**) represent the same field of view, only shown at higher magnification. Quantitative analysis confirmed these observations (**g**,**h**). Synovial lining thickness was significantly increased in OA compared to control samples (86.7 ± 18.3 µm vs. 32.8 ± 7.2 µm, ****, *p* < 0.0001). Sublining cellularity was similarly elevated in OA (668 ± 127 cells/mm^2^ vs. 304 ± 76 cells/mm^2^, ****, *p* < 0.0001). Microphotographs were captured at magnifications of 10× (**a**,**d**), 20× (**b**,**e**), and 40× (**c**,**f**). int—intima; sub—subintima; arrows indicate blood vessels. Arrows indicate representative cellular features highlighted within the tissue.

Histological examination of H&E-stained control synovium revealed regular architectural features consistent with healthy synovial tissue (Figure 1a–c). The synovial intimal lining was thin and continuous, comprising 1–2 cell layers of type A (macrophage-like) and type B (fibroblast-like) synoviocytes. The sublining layer consisted of loose connective tissue with scattered fibroblasts, small blood vessels, and adipocytes. Inflammatory cell infiltration was minimal or absent in all control samples examined. Progressive magnification from overview (Figure 1a) through intermediate (Figure 1b) to high magnification (Figure 1c) clearly demonstrated the preserved intimal-sublining architecture and low cellularity characteristic of healthy synovium.

#### 3.1.2. Synovial Inflammation and Hyperplasia in Osteoarthritis

In striking contrast, OA synovium exhibited characteristic features of chronic synovitis (Figure 1d–f). The most prominent finding was marked synovial lining hyperplasia, with the intimal layer expanded to 4–8 or more cell layers (Figure 1d,e, arrows). The sublining showed dramatically increased cellularity compared to control tissue, with dense inflammatory cell infiltration composed predominantly of lymphocytes, macrophages, and plasma cells. Focal lymphoid aggregates resembling tertiary lymphoid structures were observed in several OA samples (Figure 1f). Evidence of neovascularization was apparent, with increased numbers of small blood vessels throughout the sublining layer (Figure 1e, arrowheads). Stromal fibrosis was evident in areas of chronic inflammation. These features were consistently observed across all OA samples examined, though the severity varied among individual patients.

#### 3.1.3. Quantitative Histological Analysis

To objectively assess the histological differences between control and OA synovium, we performed quantitative morphometric analysis of two key parameters: synovial lining thickness and sublining cellularity (Figure 1g,h).

Synovial lining thickness was significantly increased in OA compared to control tissue (86.7 ± 18.3 µm vs. 32.8 ± 7.2 µm, respectively; *p* < 0.0001, unpaired *t*-test; Figure 1g). The results represent an approximately 2.6-fold increase in lining thickness, reflecting the marked synovial hyperplasia characteristic of OA synovitis. Individual patient measurements ranged from 73.8 to 96.5 µm in OA samples, compared to 28.3 to 36.4 µm in controls, with no overlap between groups.

Sublining cellularity, quantified as the number of nucleated cells per mm^2^ in standardized regions of interest, was similarly elevated in OA tissue (668 ± 127 cells/mm^2^ vs. 304 ± 76 cells/mm^2^ in controls; *p* < 0.0001, unpaired *t*-test; Figure 1h). This 2.2-fold increase in cell density reflects the extensive inflammatory cell infiltration observed in OA sublining. The increased cellularity was predominantly attributable to mononuclear inflammatory cells rather than resident fibroblasts or endothelial cells.

### 3.2. Immunofluorescence Staining of ERK 1/2

**Figure 2:**
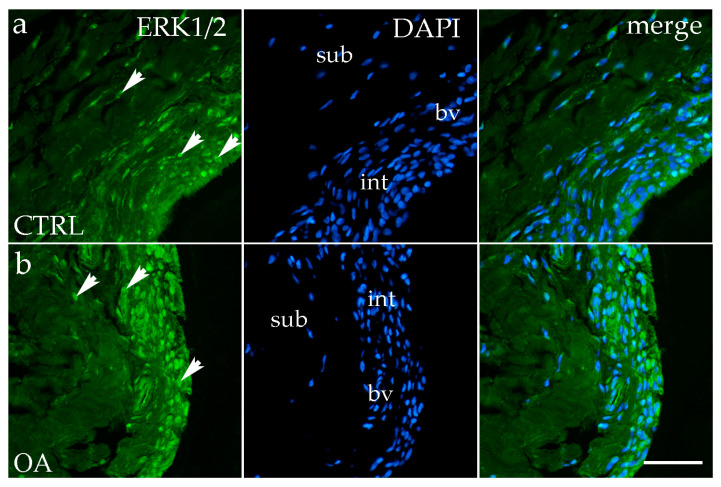
Immunofluorescence staining with Extracellular signal-regulated kinase 1/2 (ERK ½) of the synovial membrane in both sample groups. (**a**) Hip synovium of controls (CTRLs); (**b**) hip synovium in osteoarthritic group (OA); int—intima; sub—subintima; bv—blood vessel. ERK1/2-positive cells (green signal) are visible in both the intimal (arrows) and subintimal (arrows) layers of both groups examined (**a**,**b**). The far-right column (merge) shows ERK1/2 with 4′,6-diamidino-2-phenylindole (DAPI) nuclear staining. Microphotographs were captured at 40× magnification, with a scale bar of 100 μm applicable to all photos.

Immunofluorescence staining with the ERK1/2 marker demonstrated positive expression in the synovial membranes of participants with OA and healthy controls. In the CTRL and OA group, positive cells were primarily localized to the intimal blood vessels and synoviocytes (Figure 2a,b).

**Figure 3:**
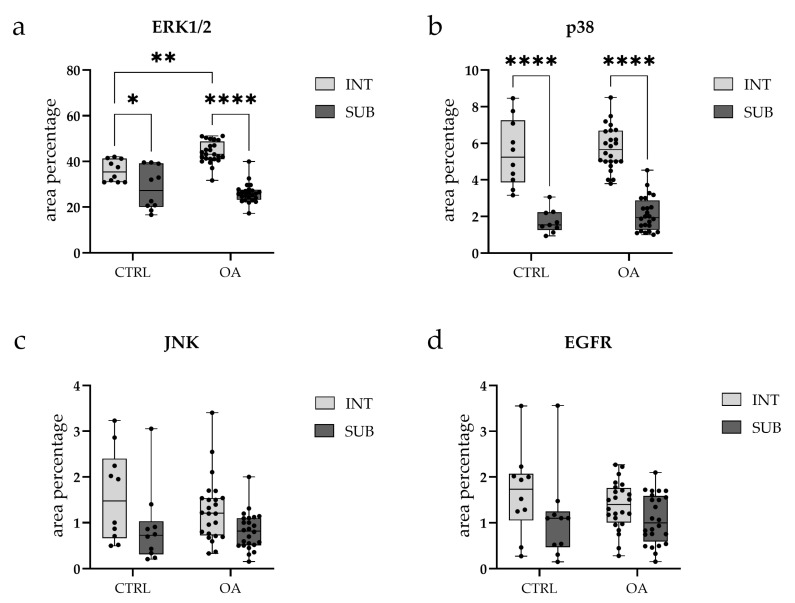
Box-and-whisker plots with overlaid individual data points showing quantitative analysis of immunofluorescence signal for (**a**) Extracellular signal-regulated kinase 1/2 (ERK1/2), (**b**) p38, (**c**) c-Jun N-terminal kinase (JNK), and (**d**) Epidermal growth factor receptor (EGFR) in synovial tissue from osteoarthritis (OA) and control (CTRL) patients. Each panel displays the percentage of positive immunostaining in two synovial subregions: the intimal layer (INT, light gray boxes) and the sublining layer (SUB, dark gray boxes). Box plots show median (horizontal line), interquartile range (box, 25th–75th percentile), and whiskers (min-max values). Individual dots represent biological replicates (CTRL, *n* = 10; OA, *n* = 24 per subregion). Statistical comparisons were performed using two-way ANOVA with Tukey’s multiple comparisons test. * *p* < 0.05, ** *p* < 0.01, **** *p* < 0.0001. Note: *Y*-axis scales differ between panels to optimize visualization of expression ranges.

Quantitative analysis demonstrated significantly higher ERK1/2 expression in the intima compared to the subintima in both CTRL (*p* < 0.05) and OA samples (*p* < 0.0001). Moreover, the intimal layer of OA samples showed a significantly greater ERK1/2-positive area compared with CTRLs (*p* < 0.01) (Figure 3a).

### 3.3. Immunofluorescence Staining of p38 MAPK

**Figure 4:**
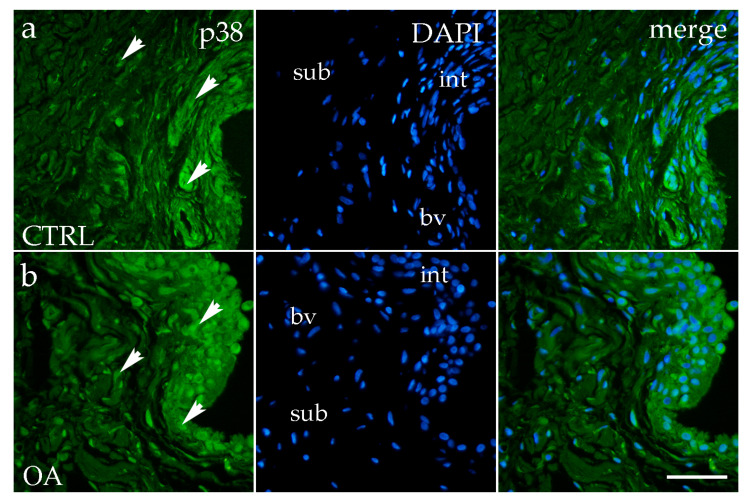
Immunofluorescence staining with p38 MAPK of the synovial membrane in both sample groups. (**a**) Hip synovium in the control (CTRL) group; (**b**) hip synovium in the osteoarthritic group (OA); int—intima; sub—subintima; bv—blood vessel. p38 MAPK cells (green signal) are visible in both the intimal (arrows) and subintimal (arrows) layers of both groups examined (**a**,**b**). The far-right column (merge) represents p38 MAPK with 4′,6-diamidino-2-phenylindole (DAPI) nuclear staining. Microphotographs were captured at 40× magnification, with a scale bar of 100 μm applicable to all photos.

Analysis of p38 MAPK expression showed positive staining in synoviocytes of the membrane in both the intima and subintima across all groups (Figure 4a,b).

The OA and CTRL samples showed no statistically significant differences. However, in each analyzed group, p38 MAPK expression was substantially higher in the intima compared to the subintima (*p* < 0.0001) (Figure 3b). Strong p38 MAPK positivity was detected in endothelial cells of the blood vessels within the intimal and subintimal layers of the CTRL group, as well as in subintimal vessels of the OA group (Figure 3b).

### 3.4. Immunofluorescence Staining of JNK

**Figure 5:**
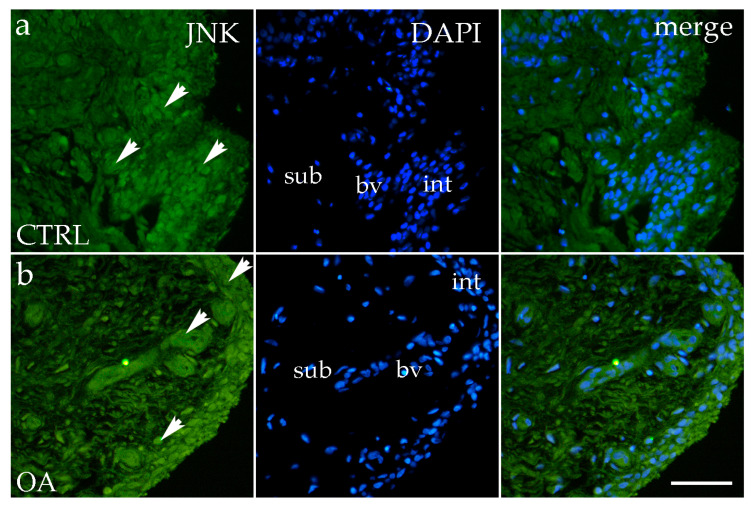
Immunofluorescence staining with c-Jun N-terminal kinase (JNK) of the synovial membrane in both groups. (**a**) Hip synovium in control (CTRL) group; (**b**) hip synovium in osteoarthritic group (OA); int—intima; sub—subintima; bv—blood vessel. JNK cells (green signal) are visible in both the intimal (arrows) and subintimal (arrows) layers of both groups examined (**a**,**b**). The far-right column (merge) represents JNK with 4′,6-diamidino-2-phenylindole (DAPI) nuclear staining. Microphotographs were captured at 40× magnification, with a scale bar of 100 μm applicable to all photos.

JNK positivity was observed in the intima and subintima in the hip of OA, as well as in the healthy CTRL group. Cells showing positivity were detected sporadically in the subintimal blood vessels of the CTRL and OA groups and intimal blood vessels of the OA group (Figure 5a,b). Synoviocytes also showed only sporadic JNK positivity (Figure 5a,b).

Overall, the intima exhibited higher JNK expression compared to the subintima, although this difference did not reach statistical significance (*p* > 0.05) (Figure 3c).

### 3.5. Immunofluorescence Staining of EGFR

**Figure 6:**
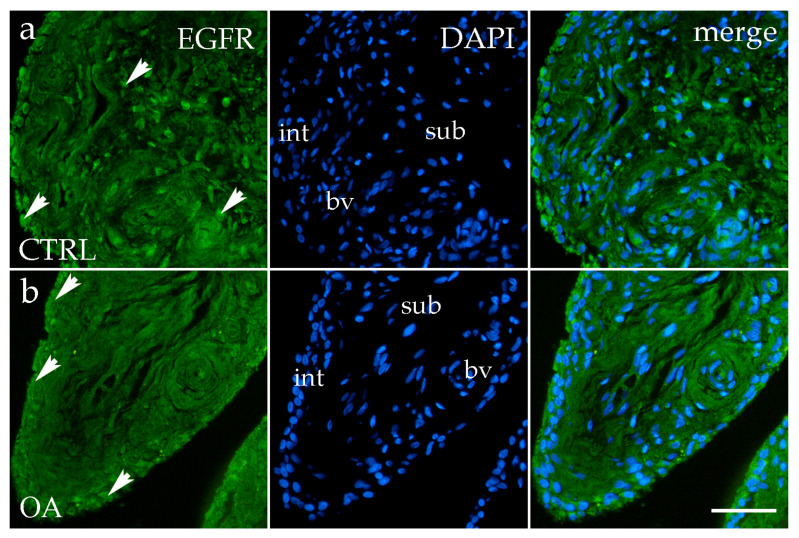
Immunofluorescence staining for Epidermal growth factor receptor (EGFR) of the synovial membrane in both sample groups. (**a**) Hip synovium in the control (CTRL) group; (**b**) hip synovium in the osteoarthritic (OA) group; int—intima; sub—subintima; bv—blood vessel. EGFR cells (green signal) are visible in both the intimal (arrows) and subintimal (arrows) layers of both groups examined (**a**,**b**). The far-right column (merge) represents EGFR with 4′,6-diamidino-2-phenylindole (DAPI) nuclear staining. Microphotographs were captured at 40× magnification, with a scale bar of 100 μm applicable to all photos.

EGFR immunoreactivity was detected in both the intimal and subintimal layers of the synovial membrane in OA and the healthy CTRL group. In the CTRL group, EGFR-positive synoviocytes and occasional subintimal cells showed moderate diffuse staining (Figure 6a). In contrast, OA samples displayed a more distinct and concentrated EGFR signal predominantly within the intimal synoviocytes, with only sporadic positivity in the subintima and perivascular regions (Figure 6b).

Quantitative analysis indicated higher EGFR expression in the intima compared to the subintima in both the OA and CTRL groups, with a more pronounced pattern in OA, although the difference did not reach statistical significance (*p* > 0.05) (Figure 3d).

### 3.6. Differential Gene Expression

Microarray gene expression data from the GSE55235 dataset (Rheumatoid arthritis and osteoarthritis: synovial tissues, Berlin cohort [58]) were analyzed to identify differential gene expression between OA and healthy CTRL synovial tissues. Particular attention was given to genes involved in the MAPK signaling pathway, including Extracellular signal-regulated kinase 1 (*MAPK3*), Extracellular signal-regulated kinase 2 (*MAPK1*), p38 (*MAPK14*), c-Jun N-terminal kinase 1 (*MAPK8*), c-Jun N-terminal kinase 2 (*MAPK9*), c-Jun N-terminal kinase 3 (*MAPK10*), and Epidermal growth factor receptor (*EGFR*). Genes showing a |log_2_(fold change)| > 1 and *p* < 0.05 were considered significantly differentially expressed.

**Figure 7:**
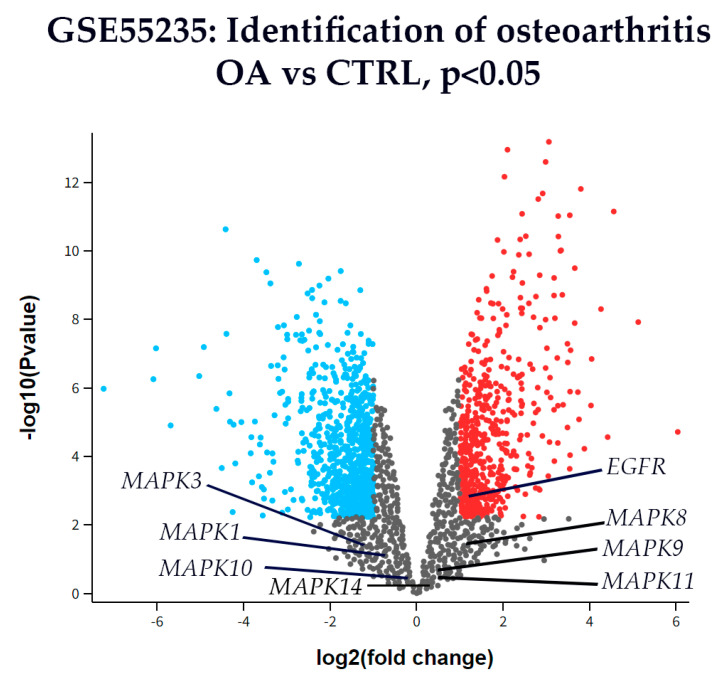
A volcano plot of differentially expressed genes in synovial tissue from OA and CTRL patients from dataset GSE55235. The *x*-axis represents the log_2_(fold change), and the *y*-axis shows the –log_10_(*p*-value). Genes with *p* < 0.01 (–log_10_(*p*) > 2) are shown in color: red for up-regulated and blue for down-regulated genes. All others are shown in gray. Among the genes analyzed, *EGFR* was the only gene significantly up-regulated in OA compared to CTRL. Other key genes from the MAPK signaling pathway—including Extracellular signal-regulated kinase 1 (*MAPK3*), Extracellular signal-regulated kinase 2 (*MAPK1*), p38 (*MAPK14*), c-Jun N-terminal kinase 1 (*MAPK8*), c-Jun N-terminal kinase 2 (*MAPK9*), c-Jun N-terminal kinase 3 (*MAPK10*) are labeled for reference, although none showed statistically significant differential expression.

Among these, only *EGFR* was significantly up-regulated in OA compared to CTRL synovial tissue (Figure 7). The expression levels of *MAPK1*, *MAPK3*, *MAPK8*, *MAPK9*, *MAPK10*, *MAPK11*, and *MAPK14* did not meet the threshold for statistical significance.

### 3.7. KEGG Pathway Enrichment Analysis Reveals Significant MAPK Pathway Activation in OA Synovium

**Figure 8:**
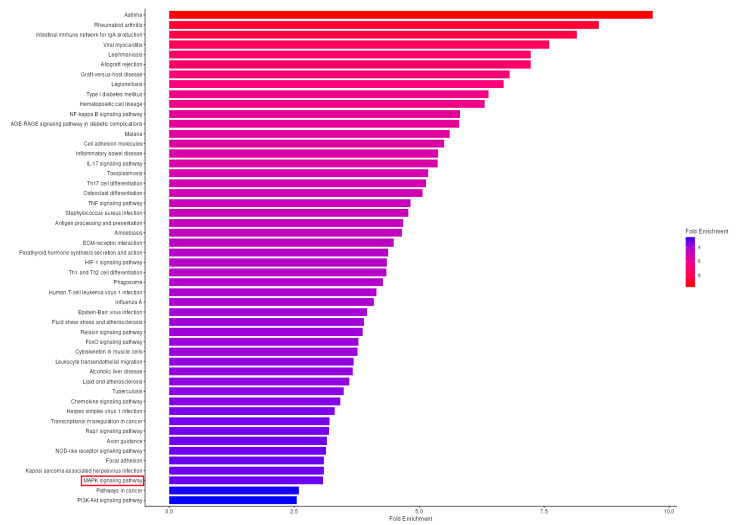
KEGG Pathway Enrichment Analysis Reveals Significant MAPK Signaling Pathway Activation in OA Synovial Tissue. KEGG pathway enrichment analysis of differentially expressed genes in osteoarthritic synovium. Horizontal bar plot showing the 50 significantly enriched KEGG pathways (FDR < 0.05) identified from 1356 differentially expressed genes (DEGs) in the GSE55235 dataset comparing OA synovial tissue (*n* = 10) to healthy controls (*n* = 10). Pathways are ranked by fold enrichment (*x*-axis), with bar length representing the magnitude of enrichment. The color gradient indicates fold enrichment values, ranging from red (highest, ~9.7-fold for Asthma) to blue (lowest, ~2.6-fold for PI3K-Akt signaling pathway and Pathways in cancer). The MAPK signaling pathway (highlighted in a red box) shows significant enrichment, with 37 genes mapped (FDR = 2.0 × 10^−8^, fold enrichment = 3.1 × 10^−19^), representing the largest number of DEGs among signaling cascades and confirming central MAPK pathway involvement in OA pathogenesis. Top enriched pathways include immune and inflammatory processes: Asthma (9.7-fold, 12 genes), Rheumatoid arthritis (8.6-fold, 32 genes, FDR = 3.1 × 10^−19^), Intestinal immune network for IgA production (8.2-fold, 16 genes), and inflammatory signaling cascades including NF-kappa B (5.8-fold, 24 genes), IL-17 (5.4-fold, 20 genes), and TNF signaling pathways (4.8-fold, 23 genes). Analysis was performed using ShinyGO v0.77 with the hypergeometric distribution test and Benjamini–Hochberg FDR correction. FDR, false discovery rate.

To characterize the biological processes and signaling networks altered in OA synovial tissue beyond individual gene expression changes, we performed KEGG pathway enrichment analysis of all 1356 differentially expressed genes identified in the GSE55235 dataset. This analysis revealed significant enrichment of 47 KEGG pathways (FDR < 0.05), with the MAPK signaling pathway emerging as a central network dysregulated in OA pathogenesis (Figure 8, Supplementary Table S1).

The MAPK signaling pathway showed significant enrichment with 37 genes mapped from the total 300 genes annotated to this pathway (FDR = 2.0 × 10^−8^, fold enrichment = 3.1). This pathway ranked among the top 50 enriched pathways and contained the largest absolute number of differentially expressed genes among signaling cascades. While individual MAPK family kinases (*MAPK1*/*ERK2, MAPK3*/*ERK1, MAPK8*/*JNK1, MAPK9*/*JNK2*, *MAPK10*/*JNK3, MAPK14*/*p38α*) did not show expression changes exceeding our statistical threshold—consistent with the known post-translational regulation of these kinases—the pathway-level analysis revealed coordinated dysregulation of 37 pathway components.

Among the pathways enriched in our analysis, several key inflammatory signaling cascades showed significant activation, including NF-kappa B signaling pathway (24 genes, FDR = 8.7 × 10^−11^, 5.8-fold enrichment), TNF signaling pathway (23 genes, FDR = 5.9 × 10^−9^, 4.8-fold enrichment), and IL-17 signaling pathway (20 genes, FDR = 1.0 × 10^−8^, 5.4-fold enrichment), all of which interact extensively with MAPK signaling to amplify inflammatory responses in OA synovium. Additionally, the Rheumatoid arthritis pathway was the most significantly enriched (32 genes, FDR = 3.1 × 10^−19^, 8.6-fold enrichment), reflecting overlapping inflammatory mechanisms in synovial pathology between OA and RA.

## 4. Discussion

### 4.1. Principal Findings and Clinical Significance

This study provides the first comprehensive characterization of MAPK pathway components—ERK1/2, p38 MAPK, JNK, and EGFR—specifically in hip OA synovium, with detailed spatial resolution between intimal and subintimal layers. Our key findings reveal that ERK1/2 demonstrates significantly elevated expression in the OA synovial intima compared to controls, accompanied by transcriptional upregulation of EGFR. In contrast, p38 MAPK, JNK, and total EGFR protein showed regional differences within synovial compartments but not between disease states. These findings suggest that ERK1/2 and EGFR may serve as biomarkers for synovial inflammation severity and represent potential therapeutic targets in hip OA, a condition with limited non-surgical treatment options.

### 4.2. ERK1/2: A Key Mediator of Synovial Inflammation in Hip OA

Our finding of significantly elevated ERK1/2 expression in the OA synovial intima represents a novel contribution to understanding the pathogenesis of hip OA. While previous studies have demonstrated increased ERK1/2 phosphorylation in knee OA cartilage [62,63,64,65], ours is the first to document this pattern specifically in the hip synovium with regional compartment analysis. The pronounced intimal localization we observed aligns with the known role of synovial lining cells in the production of inflammatory mediators and suggests that ERK1/2 activation drives synovial hyperplasia and the overproduction of inflammatory cytokines (TNF-α, IL-1β, IL-6) characteristic of OA progression [66,67,68,69].

The discordance between our protein-level findings and the absence of MAPK1/MAPK3 transcriptional changes in the GEO dataset analysis warrants careful interpretation. This pattern is consistent with the established understanding that MAPK pathway regulation occurs primarily through post-translational phosphorylation rather than transcriptional upregulation [63,70,71,72,73]. The elevated ERK1/2 protein we detected likely reflects increased pathway activation via phosphorylation, as demonstrated by Pelletier et al. in OA cartilage and by Fan et al. across OA tissue types [62,63,64,65]. Future studies employing phospho-specific antibodies (p-ERK1/2 Thr202/Tyr204) will be essential to confirm this interpretation and directly correlate phosphorylation status with disease severity markers.

An important consideration in interpreting our ERK1/2 results is the comparison with Schett et al.’s study [74], which reported elevated ERK1/2 expression in RA but not OA synovium. This apparent discrepancy can be reconciled by examining disease stage and inflammatory burden. Our OA cohort exhibited advanced synovitis, with Krenn scores comparable to those in RA (median score 2.5; 37.5% with severe inflammation), whereas Schett et al. analyzed earlier-stage OA samples with minimal synovial inflammation. This evidence suggests that ERK1/2 upregulation correlates with inflammatory intensity rather than diagnostic category per se—a hypothesis supported by the histopathological convergence of advanced OA and RA synovitis [74,75,76]. This interpretation underscores the clinical relevance of our findings: ERK1/2 expression may serve as a biomarker to identify OA patients with aggressive synovial inflammation who may benefit from targeted MAPK inhibition.

### 4.3. p38 MAPK: Conserved Regional Expression Without Disease-Specific Changes

We observed significantly higher p38 MAPK expression in the synovial intima compared with the subintima in both OA and control groups, but no difference between disease states. This regional pattern suggests a physiological role for p38 MAPK in normal synovial lining function, potentially related to baseline inflammatory surveillance and cellular stress responses [77,78,79,80]. The absence of disease-associated differences in total protein levels contrasts with studies reporting elevated p38 phosphorylation in OA tissues [81,82]. Still, it aligns with Shi et al.’s findings in a rabbit OA model, where total p38 remained unchanged despite increased phosphorylation [83].

The methodological focus of previous OA research on cartilage rather than synovium limits direct comparison with our findings [81,82,84,85]. The few synovium-focused studies have primarily examined RA, where Schett et al. and Korb et al. demonstrated enhanced p38 expression in synovial endothelial and intimal cells, driven by TNF-α and IL-1 [74,75,80]. The fact that we observed similar p38 levels in advanced OA (with high Krenn scores) and controls suggests that p38 activation—rather than expression—is the critical pathogenic mechanism. This interpretation is consistent with the known biology of p38 MAPK, where stimulus-induced phosphorylation at Thr180/Tyr182 determines pathway activation independently of total protein abundance [27,32,33,34].

### 4.4. JNK: Context-Dependent Activation in Synovial Inflammation

JNK immunoreactivity was detected throughout the synovial membrane without statistically significant differences between OA and control groups or between regional compartments. This finding diverges from studies reporting elevated phospho-JNK (p-JNK) in OA cartilage [62,86,87,88]. Still, it resonates with Loeser et al.’s demonstration that JNK’s role in OA is complex and context-dependent [89]. Their finding that *JNK* deletion exacerbated age-related cartilage degeneration suggests that basal JNK activity may be protective, with pathogenic effects emerging only under specific inflammatory conditions.

The tissue-specific nature of JNK signaling likely explains some inconsistencies in the literature. While cartilage studies emphasize JNK’s contribution to MMP-mediated matrix degradation [90,91], synovium-focused investigations reveal more nuanced roles. Görtz et al. found that TNF-α predominantly activated p38 and ERK—but not JNK—in synovial cells [75], while Launay et al. reported strong p-JNK only in RA synovium [92]. Our synovial findings align more closely with Görtz’s TNF-driven synovitis model, suggesting that JNK activation in hip OA synovium may require specific inflammatory triggers beyond the basal TNF-α/IL-1β milieu.

An important caveat is that our assessment of total JNK protein cannot capture activation dynamics. As with ERK1/2 and p38, JNK pathway activity is governed by phosphorylation (Thr183/Tyr185) rather than protein abundance. The sporadic JNK positivity we observed may indicate low-level constitutive expression, with functional activation occurring in specific cellular subpopulations or microanatomical niches that our sampling approach did not capture.

### 4.5. EGFR: Dual Roles and Stage-Dependent Expression in OA

EGFR emerged as the only MAPK pathway component showing significant transcriptional upregulation in our GEO dataset analysis, while protein-level expression demonstrated intimal predominance without reaching statistical significance between groups. This pattern suggests active transcriptional regulation of EGFR in OA synovium, potentially reflecting compensatory responses to inflammatory stress or involvement in synovial remodeling [44,45,46,47,48].

The interpretation of EGFR’s role in OA is complicated by its well-documented dual functionality: chondroprotective in early disease and catabolic in advanced stages [45,46,47,93]. Sun et al. reported reduced EGFR protein and mRNA in damaged cartilage [48], contrasting with our observation of elevated EGFR transcripts in OA synovium. This discrepancy likely reflects tissue-specific differences (synovium vs. cartilage) and the distinct roles EGFR plays in these compartments. In cartilage, EGFR maintains chondrocyte viability and matrix integrity; in synovium, EGFR upregulation may drive the production of inflammatory mediators and contribute to synovitis [47,48,94].

The stage-dependent effects of EGFR must also be considered when comparing studies. Wei et al. demonstrated that phospho-EGFR decreases in early OA but reappears in late-stage disease [45], while our cohort consisted of advanced hip OA patients undergoing arthroplasty. The elevated EGFR expression in our OA samples may therefore represent late-stage reactivation associated with advanced synovitis. Supporting this interpretation, Swanson et al. and Li et al. found increased EGFR expression in RA synovium [95,96], and our OA cohort’s high Krenn scores indicate an inflammatory severity comparable to that in RA. This evidence suggests that EGFR upregulation parallels inflammatory burden across arthritides, regardless of underlying etiology.

### 4.6. Pathway-Level Integration: KEGG Analysis Reveals Coordinated MAPK Network Dysregulation

Our KEGG pathway enrichment analysis provided critical context for interpreting individual gene expression patterns. Despite minimal changes in MAPK transcripts (*MAPK1*, *MAPK3*, *MAPK8*, *MAPK9*, *MAPK10*, and *MAPK14*), the MAPK signaling pathway as a whole showed significant enrichment (37 genes, FDR = 2.0 × 10^−8^), demonstrating coordinated dysregulation of upstream activators, downstream effectors, and regulatory feedback components. The considerable upregulation of EGFR—a key upstream activator—combined with enrichment of inflammatory cascades (NF-κB, TNF, IL-17 pathways) illustrates the extensive crosstalk through which MAPK signaling amplifies synovial inflammation in OA.

This systems-level perspective reconciles the apparent contradiction between unchanged kinase transcript levels and clear evidence of pathway activation at the protein level. The enrichment of 37 MAPK pathway components indicates that OA synovium exhibits broad transcriptional reprogramming affecting pathway regulators and effectors, even while core kinase expression remains stable. This pattern is entirely consistent with MAPK biology, in which pathway flux is controlled post-translationally through phosphorylation cascades initiated by upstream receptors such as EGFR [10,11,12,13,14,15].

### 4.7. Limitations and Future Directions

Several limitations merit acknowledgment. First and most importantly, we assessed total protein expression rather than phosphorylation status—the definitive indicator of MAPK pathway activation. While our regional distribution patterns likely reflect underlying activation domains, as supported by prior phosphorylation studies in OA tissues [62,63,64,65], future investigations must employ phospho-specific antibodies (p-ERK1/2, p-p38, p-JNK, p-EGFR) to directly measure kinase activation across synovial subregions and correlate with disease severity, inflammatory profiles, and clinical outcomes.

Second, our control tissue was obtained from femoral neck fracture patients rather than healthy elective donors (due to ethical constraints), potentially introducing confounding effects of acute trauma. However, we applied strict exclusion criteria (no radiographic OA, negative inflammatory serology) and observed minimal inflammation in control samples (median Krenn score 0.5), supporting their validity as comparators for chronic OA synovitis.

Third, the relatively small sample size (*n* = 10 controls, *n* = 24 OA) and focus on advanced hip OA patients undergoing arthroplasty limits generalizability to earlier disease stages or knee OA. The joint-specific biomechanical and inflammatory characteristics of hip vs. knee OA warrant dedicated investigation in each articulation [1,4,6].

Finally, the cross-sectional design precludes assessment of temporal dynamics. Longitudinal studies tracking changes in MAPK phosphorylation during disease progression, ideally with paired synovial and cartilage samples, would provide mechanistic insights into the sequence of pathway activation events and their relationship to structural joint damage.

### 4.8. Clinical and Translational Implications

Our identification of ERK1/2 and EGFR as differentially regulated MAPK components in hip OA synovium has direct translational relevance. ERK1/2, given its significant upregulation in OA intima and its established role in synovial inflammation [66,67,68,69], represents a promising therapeutic target. Several naturally derived compounds—including vicenin-3, formononetin, and garlic-derived exosomes—have demonstrated ERK1/2 inhibitory activity in preclinical OA models [70,71,72], warranting clinical evaluation. Additionally, ERK1/2 expression levels may serve as a biomarker for stratifying OA patients by synovitis severity, enabling personalized treatment approaches.

EGFR targeting requires more nuanced consideration, given its dual protective and pathogenic roles depending on disease stage and tissue context [45,46,47,70,71,72,93]. Selective modulation—rather than complete inhibition—may be necessary to harness EGFR’s chondroprotective effects while limiting its contribution to synovial inflammation in advanced disease. The development of tissue-specific EGFR modulators or stage-tailored therapeutic strategies represents an important avenue for future investigation.

## 5. Conclusions

This study establishes ERK1/2 as a key mediator of synovial inflammation in hip OA, demonstrating region-specific upregulation in the intimal layer where inflammatory cytokine production is concentrated. Combined with transcriptional upregulation of EGFR, these findings identify specific MAPK pathway nodes as potential therapeutic targets for hip OA synovitis. The discordance between protein and mRNA levels of MAPKs underscores the critical importance of assessing phosphorylation status in future studies to definitively characterize pathway activation. Our results provide a foundation for developing targeted interventions addressing synovial inflammation in hip OA—a condition with limited treatment options beyond joint replacement—and support the exploration of ERK1/2 inhibitors and EGFR modulators as novel therapeutic strategies.

## Figures and Tables

**Table 1:** Clinical, radiological, and pathohistological features of the study groups (*n* = 34).

	Control Group	Krenn Score of Synovitis in OA (0–2)	Krenn Score of Synovitis in OA ≥ 3	* *p*-Value
Age in years: Median ± IQR	72.9 (64–77)	73.3 (70–82)	69.9 (58–82)	0.884
Sex: Male/Female	(6/4)	(7/5)	(6/6)	0.732
BMI (Median ± IQR, kg/m^2^)	25.87 (23.97–26.6)	24.7 (23.25–25.82)	26.7 (25.5–29.43)	0.054
K-L grade (median ± IQR)	0.5 (0–1)	2 (2–2)	4 (3–4)	<0.0001
Krenn Score (Median ± IQR)	0 (0–0)	6.4 (5.6–9)	9 (7–9)	<0.0001
HHS (Median ± IQR)	-	48.7 (43.58–56.8)	41 (33.48–49.6)	0.272
VAS (Median ± IQR)	-	6 (4.6–6.8)	6 (5–7)	0.784
WOMAC (Median ± IQR)	-	46.2 (40.2–56.4)	47.3 (36.1–55.3)	0.918

IQR (interquartile range), OA (osteoarthritis), BMI (body mass index), K-L grade (Kellgren–Lawrence grading scale), HHS (Harris Hip Score), VAS (visual analogue scale), WOMAC (The Western Ontario and McMaster Universities Osteoarthritis Index); * *p* < 0.05, Kruskal–Wallis test.

**Table 2:** Immunofluorescence antibodies used in the study.

Antibodies		Catalog Number	Host	Dilution	Source
Primary	p44/42 MAPK (Erk1/2) (137F5)	4695S	Rabbit	1:300	Cell Signaling Technology, Inc. (CST) Danvers, MA, USA
	EGF Receptor (D38B1) XP^®^	4267S	Rabbit	1:100	
	p38 MAPK Antibody	9212S	Rabbit	1:100	
	SAPK/JNK Antibody	9252S	Rabbit	1:100	
Secondary	Alexa Fluor^®^ 488 AffiniPure^®^ Donkey Anti-Rabbit IgG (H + L)	711-545-152	Donkey	1:300	Jackson ImmunoResearch Laboratories, Inc., West Grove, PA, USA

## Data Availability

The data supporting the findings of this study are available from the corresponding author upon reasonable request. Gene expression data for *MAPK1*, *MAPK3*, *MAPK8*, *MAPK9*, *MAPK10*, *MAPK11*, and *EGFR* were obtained from publicly available databases, including the Gene Expression Omnibus (GEO) database (NCBI). Specifically, data from the GSE55235 series (Rheumatoid arthritis and osteoarthritis: synovial tissues—Berlin dataset [58]) were utilized.

## Supplementary Materials

The following supporting information can be downloaded at: https://www.mdpi.com/article/10.3390/biomedicines13122992/s1, Table S1: KEGG Pathway Enrichment Analysis of Differentially Expressed Genes in OA Synovial Tissue.

## Abbreviations

The following abbreviations are used in this manuscript:

ADAMTS5	A Disintegrin and Metalloproteinase with Thrombospondin Type 1 Motif 5
ANOVA	analysis of variance
anti-CCP	anti-cyclic citrullinated peptides
BMI	body mass index
bv	blood vessel
CTRLs	controls
DAPI	4′,6-diamidino-2-phenylindole
DEGs	differentially expressed genes
DRP1	Dynamin-Related Protein 1
EGFR	Epidermal Growth Factor Receptor
ERK 1/2	Extracellular Signal-Regulated Kinase 1/2
FDR	False discovery rate
GDE	garlic-derived exosomes
GEO	Gene Expression Omnibus
H&E	Hematoxylin–eosin
HHS	Harris Hip Score
HSS	higher synovitis score group
ICAM-1	Intercellular Adhesion Molecule 1
ICC	intraclass correlation coefficient
IHC	Immunohistochemistry
IL-1β	Interleukin-1 beta
INT	intima
IQR	interquartile range
JNK	c-Jun N-terminal kinase
KEGG	Kyoto Encyclopedia of Genes and Genomes
K-L	Kellgren–Lawrence
MAPK	mitogen-activated protein kinase
MMP-1	Matrix Metalloproteinase-1
NCBI	National Center for Biotechnology Information
mRNA	messenger RNA
OA	Osteoarthritis
p38/SAPK	p38 Mitogen-Activated Protein Kinase/stress-activated protein kinase
PBS	phosphate-buffered saline
qRT-PCR	quantitative real-time reverse transcription polymerase chain reaction
RA	rheumatoid arthritis
RF	rheumatoid factor
RNAseq	RNA sequencing
ROI	regions of interest
SD	standard deviations
SUB	subintima
TNF-α	tumor necrosis factor alpha
VAS	visual analog scale
WOMAC	Western Ontario and McMaster Universities Arthritis Index
