# Supplementary material for: MAPK Pathway Activation Patterns in the Synovium Reveal ERK1/2 and EGFR as Key Players in Osteoarthritis
Source: Biomedicines. 2025 Dec 5;13(12):2992. doi: 10.3390/biomedicines13122992 (PMC12730786; doi:10.3390/biomedicines13122992)
Supplement: Supplementary file 1 [file biomedicines-13-02992-s001.zip › biomedicines-3982777-supplementary.pdf]

**Supplementary Table S1.** KEGG Pathway Enrichment Analysis of Differentially Expressed Genes in OA Synovial Tissue. Top 50 list of significantly enriched KEGG pathways identified from differential gene expression analysis of GSE55235 dataset. The table presents 47 KEGG pathways showing significant enrichment (FDR < 0.05) among the 1356 differentially expressed genes (DEGs) identified when comparing osteoarthritic synovial tissue (n=10) to healthy control synovium (n=10). Pathways are ranked by fold enrichment in descending order. Analysis was performed using ShinyGO v0.77 (http://bioinformatics.sdstate.edu/go/) with hypergeometric distribution test for statistical assessment.

| Enrichment FDR | nGenes | Pathway Genes | Fold Enrichment | Pathways (click for details) |
|---|---|---|---|---|
| 1.7E-08 | 12 | 31 | 9.7 | Asthma |
| 3.1E-19 | 32 | 93 | 8.6 | Rheumatoid arthritis |
| 1.0E-09 | 16 | 49 | 8.2 | Intestinal immune network for IgA production |
| 1.0E-11 | 21 | 69 | 7.6 | Viral myocarditis |
| 1.0E-11 | 22 | 76 | 7.2 | Leishmaniasis |
| 1.8E-06 | 11 | 38 | 7.2 | Allograft rejection |
| 1.1E-06 | 12 | 44 | 6.8 | Graft-versus-host disease |
| 4.8E-08 | 15 | 56 | 6.7 | Legionellosis |
| 5.1E-06 | 11 | 43 | 6.4 | Type I diabetes mellitus |
| 9.8E-12 | 25 | 99 | 6.3 | Hematopoietic cell lineage |
| 8.7E-11 | 24 | 103 | 5.8 | NF-kappa B signaling pathway |
| 2.1E-10 | 23 | 99 | 5.8 | AGE-RAGE signaling pathway in diabetic complications |
| 1.9E-05 | 11 | 49 | 5.6 | Malaria |
| 1.7E-14 | 35 | 159 | 5.5 | Cell adhesion molecules |
| 2.0E-06 | 14 | 65 | 5.4 | Inflammatory bowel disease |
| 1.0E-08 | 20 | 93 | 5.4 | IL-17 signaling pathway |
| 1.8E-09 | 23 | 111 | 5.2 | Toxoplasmosis |
| 4.5E-09 | 22 | 107 | 5.1 | Th17 cell differentiation |
| 1.1E-10 | 27 | 133 | 5.1 | Osteoclast differentiation |
| 5.9E-09 | 23 | 119 | 4.8 | TNF signaling pathway |
| 3.8E-07 | 18 | 94 | 4.8 | Staphylococcus aureus infection |
| 4.4E-06 | 15 | 80 | 4.7 | Antigen processing and presentation |
| 2.7E-07 | 19 | 102 | 4.7 | Amoebiasis |
| 3.4E-06 | 16 | 89 | 4.5 | ECM-receptor interaction |
| 3.2E-07 | 20 | 114 | 4.4 | Parathyroid hormone synthesis secretion and action |
| 6.7E-07 | 19 | 109 | 4.4 | HIF-1 signaling pathway |
| 5.1E-06 | 16 | 92 | 4.3 | Th1 and Th2 cell differentiation |
| 6.6E-09 | 26 | 152 | 4.3 | Phagosome |
| 1.0E-11 | 37 | 223 | 4.1 | Human T-cell leukemia virus 1 infection |
| 4.5E-09 | 28 | 171 | 4.1 | Influenza A |
| 8.4E-10 | 32 | 202 | 4 | Epstein-Barr virus infection |
| 5.3E-07 | 22 | 141 | 3.9 | Fluid shear stress and atherosclerosis |
| 1.9E-06 | 20 | 129 | 3.9 | Relaxin signaling pathway |
| 2.5E-06 | 20 | 132 | 3.8 | FoxO signaling pathway |
| 4.5E-10 | 35 | 232 | 3.8 | Cytoskeleton in muscle cells |
| 2.1E-05 | 17 | 115 | 3.7 | Leukocyte transendothelial migration |
| 2.3E-06 | 21 | 143 | 3.7 | Alcoholic liver disease |
| 1.0E-08 | 31 | 215 | 3.6 | Lipid and atherosclerosis |
| 6.2E-07 | 25 | 179 | 3.5 | Tuberculosis |

| | | | | |
|---|---|---|---|---|
| 5.3E-07 | 26 | 190 | 3.4 | Chemokine signaling pathway |
| 2.3E-06 | 24 | 181 | 3.3 | Herpes simplex virus 1 infection |
| 2.5E-06 | 25 | 195 | 3.2 | Transcriptional misregulation in cancer |
| 1.1E-06 | 27 | 211 | 3.2 | Rap1 signaling pathway |
| 7.8E-06 | 23 | 182 | 3.2 | Axon guidance |
| 8.4E-06 | 23 | 183 | 3.1 | NOD-like receptor signaling pathway |
| 4.4E-06 | 25 | 202 | 3.1 | Focal adhesion |
| 6.9E-06 | 24 | 194 | 3.1 | Kaposi sarcoma-associated herpesvirus infection |
| 2.0E-08 | 37 | 300 | 3.1 | MAPK signaling pathway |
| 2.6E-09 | 55 | 529 | 2.6 | Pathways in cancer |
| 1.9E-06 | 37 | 362 | 2.6 | PI3K-Akt signaling pathway |
